# The Antimicrobial Effects of Myrosinase Hydrolysis Products Derived from Glucosinolates Isolated from *Lepidium draba*

**DOI:** 10.3390/plants13070995

**Published:** 2024-03-30

**Authors:** Zoltán Polozsányi, Helena Galádová, Michal Kaliňák, Martin Jopčík, Barbora Kaliňáková, Albert Breier, Martin Šimkovič

**Affiliations:** 1 Institute of Biochemistry and Microbiology, Faculty of Chemical and Food Technology, Slovak University of Technology in Bratislava, Radlinského 9, 812 37 Bratislava, Slovakia; 2 Central Laboratories, Faculty of Chemical and Food Technology, Slovak University of Technology in Bratislava, Radlinského 9, 812 37 Bratislava, Slovakia; 3 Institute of Plant Genetics and Biotechnology, Plant Science and Biodiversity Center, Slovak Academy of Sciences, Akademická 969, 949 01 Nitra, Slovakia; 4 Institute of Molecular Physiology and Genetics, Centre of Biosciences, Slovak Academy of Sciences, Dúbravská Cesta 9, 845 05 Bratislava, Slovakia

**Keywords:** glucoraphanin, sinalbin, *Lepidium draba*, myrosinase (β-thioglucosidase, EC 3.2.1.147), antimicrobial activity

## Abstract

*Lepidium draba* (hoary cress) is a perennial plant belonging to the *Brassicaceae* family that produces two dominant glucosinolates (GLSs): glucoraphanin (GRN) and sinalbin (SBN). They represent the stored form, which is converted upon the myrosinase (Myr) hydrolysis activity to active compounds, mainly isothiocyanates (ITCs) such as sulforaphane (SFN) or *p*-hydroxybenzyl isothiocyanate (pHBITC). Research on ITCs that have proven anticancer, antimicrobial, and chemoprotective properties is usually conducted with pure commercially available compounds. However, these are chemically reactive, making it difficult to use them directly for preventive purposes in dietary supplements. Efforts are currently being made to prepare dietary supplements enriched with GLS and/or Myr. In this study, we report a simple but efficient chromatographic procedure for the isolation and purification of GLSs from MeOH extract from hoary cress based on a combination of ion exchange and gel permeation chromatography on DEAE-Sephadex A-25 and Sephadex LH-20. To obtain the Myr required for efficient hydrolysis of GLSs into antibacterial ITCs, we developed a rapid method for its extraction from the seeds of *Lepidium sativum* (garden cress). The yields of GLSs were 22.9 ± 1.2 mg GRN (purity 96%) and 10.4 ± 1.1 mg SBN (purity 92%) from 1 g of dry plant material. Both purified GLSs were used as substrates for the Myr. Analysis of the composition of hydrolysis products (HPs) revealed differences in their hydrolysis rates and in the degree of conversion from GLSs to individual ITCs catalyzed by Myr. When GRNs were cleaved, SFNs were formed in an equimolar ratio, but the formation of pHBITCs was only half that of cleaved SBNs. The decrease in pHBITC content is due to its instability compared to SFN. While SFN is stable in aqueous media during the measurement, pHBITC undergoes non-enzymatic hydrolysis to *p*-hydroxybenzyl alcohol and thiocyanate ions. Testing of the antimicrobial effects of the HPs formed from GRN by Myr under premix or in situ conditions showed inhibition of the growth of model prokaryotic and eukaryotic microorganisms. This observation could serve as the jumping-off point for the design of a two-component mixture, based on purified GLSs and Myr that is, usable in food or the pharmaceutical industry in the future.

## 1. Introduction

Glucosinolates (GLSs) are β-thioglucoside secondary plant metabolites commonly present in plants of the *Brassicaceae* family. All known GLSs contain a basic thioglugoside core structure formed by a hydrophilic β-D-glucopyranose unit linked by a NO-sulfated thiohydroximate linker to an aglycone moiety, which exhibits wide structural variability, including both aliphatic and aromatic structures. Around 130 different GLSs have been identified [1]. Precursors of GLS biosynthesis are amino acids. The entire GLS biosynthetic pathway is divided into three phases: I. amino acid chain elongation (in chloroplasts); II. basic structure synthesis (in cytosol); and III. secondary modifications of parent GLSs, which provide high structural variability [2]. These modifications, e.g., oxidation, hydroxylation, methoxylation, desaturation, sulfation, or glycosylation, take place in the different parts of plants and are dependent on the developmental stage [3,4,5]. The biosynthesis of GLSs is regulated at the transcription level (R2R3-MYB transcription factor gene family) [6,7]. Since GLSs are sulfur-containing metabolites, their quantity and biosynthesis are affected by the availability of sulfur in the environment and additionally regulated at the transcriptional level (transcription factor SLIM1) [8,9,10]. GLSs themselves are only negligibly biologically active. Their activation is caused by enzymatic hydrolysis by myrosinase (Myr, β-thioglucosidase, EC 3.2.1.147) (glucosinolate–myrosinase system) [11]. This system is a highly effective defense mechanism for cruciferous plants [12]. Since GLSs and Myr are localized in isolated plant tissue structures, this defense system is activated after physical damage to plant cells by herbivores, insects, or phytopathogens when GLS substrates come into contact with Myr [13,14]. Hydrolysis of GLSs results in the release of both D-glucose and the unstable aglycone, which spontaneously decomposes into several compounds with different toxicities. These include isothiocyanates (ITCs), nitriles, epithionitriles, and thiocyanates, depending on the reaction conditions (e.g., pH, the presence of Fe^2+^ ions, and epithiospecifier protein) and the nature of the GLSs side chain [15,16,17,18,19]. Of the above GLS products, the biological potencies of ITCs, which are reactive electrophilic compounds that react with nucleophilic functional groups (-SH, -NH_2_, and -OH) present on small and large molecules [20], are currently the most studied.

Although the antibacterial [21] and antifungal [22] activities of ITCs have been known for more than 50 years and seem to be correlated with the lipophilicity of the derivatives [21], the exact recognition of all the mechanisms involved in their effectiveness has not yet been satisfactorily elucidated. This is because ITCs can attack nucleophilic groups (-SH, -NH_2_, and -OH) of practically all biomolecules, including phospholipids, carbohydrates, proteins, and many metabolites [20]. In addition to inhibiting cell growth [23,24], they also affect the activity of several enzymes [25], regulatory mechanisms, e.g., quorum sensing [23], and even the function of transcription factors, e.g., Nrf2 [26] and their effects also induce morphological changes [27]. Recent studies have indicated that the synergic effect of GLS HPs and antibiotics could provide a therapeutic solution in the fight against antibiotic-resistant microbial infections [28,29,30,31]. Some ITCs, including sulforaphane (SFN), if they attain sufficient amounts in the bodies of mammals through the consumption of fresh vegetables from the cruciferous family, have significant health-promoting effects in the prevention of diseases such as neoplastic, cardiovascular, neurodegenerative, and metabolic diseases [32,33,34,35]. The chemoprotective and anticancer effects of ITCs were also attributed to their effects on Keap1-Nrf2, PI3K/AKT/mTOR, MAPK/EKR/JNK, and NF-κB signaling pathways [33]. SFN, which arises from glucoraphanin (GRN), is the most often mentioned compound in these contexts. It has been shown that SFN can inhibit cancer development through multilevel interference with cellular processes, e.g., by inducing the expression of genes encoding enzymes of the II phases [36] but not the I phase [37] of detoxification, reducing oxidative stress, and maintaining the balance of antioxidative and prooxidative events in the cell as indirect antioxidants [38]. In tumor-transformed cells, it can cause the inhibition of proliferation, entry of cells into apoptosis, or autophagy [39,40,41,42,43].

Currently, nutritional supplements, such as extracts from broccoli or freeze-dried broccoli sprouts [44], are most often offered as rich sources of GLSs. Ex vivo studies revealed that the bioavailability of GLSs in the gastrointestinal tract is not only dependent on the initial quantity of GLSs in the plant material but rather on the release of GSLs from the material and the continuing cleavage of GLSs during digestion [45]. However, the Myr activity in nutritional preparations is either very low or null, and the conversion of GLSs to HPs, executed by the gut microbiota, is not so effective for GLSs and HPs to become bioavailable [46,47]. Addressing these limitations could involve creating a two-component preparation containing purified GLSs and enriched with isolated Myr. Such a nutritional supplement could significantly improve the ability to conduct population-based studies in cancer chemoprevention [48]. Therefore, the presented study was focused on the development of a procedure for the laboratory preparation of purified GRN from *Lepidium draba* (hoary cress, details about the plant species see [49,50]) and an enzyme preparation with Myr activity from the seeds of *Lepidium sativum* (garden cress; for details about the plant species, see [49,51]) and analytical verification of the products of the transformation of GRN to SFN catalyzed by the Myr preparation. Another goal was to verify the antimicrobial effects of GRN hydrolysis products by the action of Myr activity under premix conditions.

## 2. Results

### 2.1. Purification of GRN

In a series of preliminary experiments, we identified hoary cress as a suitable source of GLS among several plants. In addition to the dominant GRN, it contained a greater amount of SBN [52]. The main problem in the initial stage of GRN purification was the presence of plant color pigments that coextract with GLS in 80% (*v*/*v*) MeOH. Such extracts could not be directly applied to chromatographic columns because the phytopigments bound to and stained the chromatographic matrices, thereby damaging them. Complete removal of color pigments and an increase in the purity of methanol extracts were achieved by two-step adsorption/extraction using activated charcoal.

**Figure 1:**
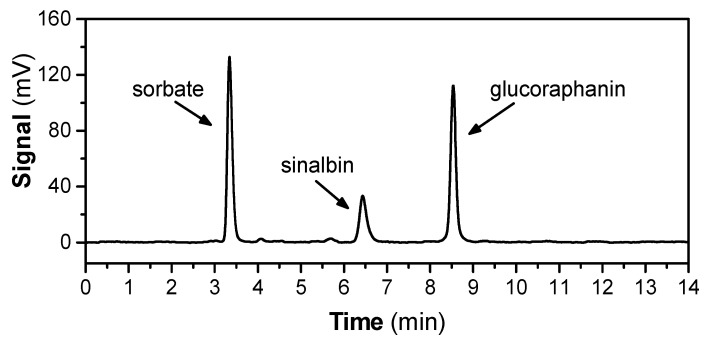
Hydrophilic interaction liquid chromatography (HILIC) chromatogram showing the GLSs profile of hoary cress leaves. The GLSs profile represents the fraction obtained from extraction in 80% MeOH, decolorization on activated charcoal, followed by ion-exchange chromatography on DEAE Sephadex A 25 (see Section 4.5). Potassium sorbate was used as internal standard for quantitative analysis. The data are representative of several extracts obtained from hoary cress leaves or flowers that were processed in the same way, and the results are summarized in Table 1.

Analysis of the fractions obtained after the ion exchange chromatography (IEC; see Section 4.3 on DEAE Sephadex A 25) using high-pressure hydrophilic interaction liquid chromatography (HILIC on EC 150/4.6 Nucleodur HILIC 5 μm column containing sulfoalkylbetaine zwitterionic functional groups; for detail, see Section 4.5) showed the presence of both GRN and SBN. Some minor and unknown substances were also present in material eluted from the IEC column, whose contents were negligible, and they were not identified. SBN (with lower hydrophilicity) was eluted from the HILIC column with a shorter retention time (6.44 min) than more hydrophilic GRN (8.54 min), as shown in Figure 1. By contrast, GRN interacted intensively with the sulfoalkylbetaine zwitterionic ligand of the column.

The elution profiles of GLSs in extracts obtained from leaves and flowers were similar. The total yield of GLS obtained after IEC and the amount of both dominant GLS present in the leaves or flowers of hoary cress are summarized in Table 1. In the leaves, the GRN content reached 28.7 ± 2.4 mg/g dry plant material and was twice as high as the SBN content 14.7 ± 0.5 mg/g dry plant material. Almost a 4.2 times higher amount of GRN compared to SBN was observed in the flower. The fractions after IEC were lyophilized, but due to high hygroscopicity, the lyophilizates had a rather pasty than solid consistency.

**Figure 2:**
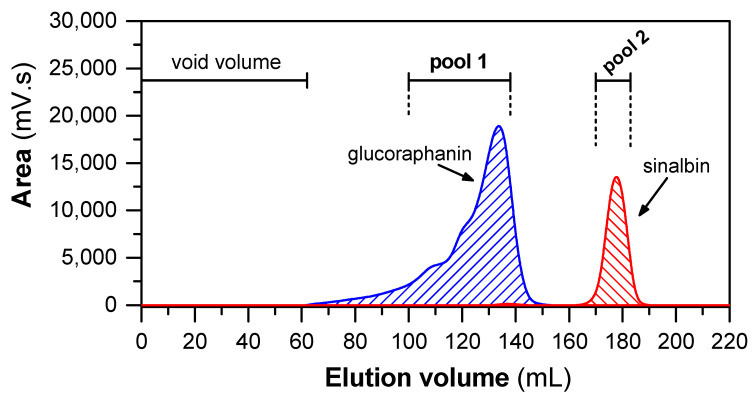
Elution profile of GLSs separated by GPC on Sephadex LH-20 column. The 4 mL of sample (with 400 mg of fraction obtained after IEC) was loaded into column (109 cm × 1.5 cm). The elution was isocratic, using 80% (*v*/*v*) MeOH as eluent at flow rate of 1 mL/min. Subsequently, the collected fractions were analyzed by HILIC and pooled according to the content of GRN and SBN.

Since the material obtained from IEC contained a mixture of both GLSs, they were separated in the next step by gel permeation chromatography (GPC; for protocol, see Section 4.4) on Sephadex LH-20. GPC on this column is based on both hydrophilic and lipophilic properties of the Sephadex LH-20 matrix [53]. Figure 2 shows a typical GPC elution profile of a sample containing GLSs obtained from hoary cress leaves. The pooled fractions from the first chromatographic peak contained GRN with 96% purity, and the pooled fractions from the second chromatographic peak contained SBN with 92% purity (see Section 4.5).

Both GLSs were further analyzed by ^1^H-NMR and MALDI-TOF to prove their identity and confirm their structure (see Section 2.2). The obtained yields after purification by both chromatographic steps were 22.9 ± 1.2 mg/g (of dry plant material) and 10.4 ± 1.1 mg/g (of dry plant material) for GRN and SBN, respectively. However, SBN showed high hygroscopicity and rapidly absorbed moisture from the environment, which burdened the accuracy of its yield determination.

Alternatively to the described purification method, it was possible to use preparative thin-layer chromatography (TLC; see Figure S1A in Supplementary Material). Even though we successfully separated GRN from SBN, the purity of the products was 90% for GRN and 82% for SBN, which was less satisfactory. The products contained traces of acetate, MeOH, and formate, which were detected by ^1^H-NMR analysis (Figure S1B in Supplementary Material).

### 2.2. Characterisation of GRN and SBN by ^1^H-NMR and MS/MS Analysis

First, we analyzed the obtained products by proton magnetic resonance. The glucosyl group of GRN contains an anomeric proton (H1′ with 5.04 ppm), which is well resolved in ^1^H-NMR (Table 2), and its coupling constant of 9.9 Hz reveals a β-anomeric configuration [54,55,56]. The remaining protons of the glucose group have the following signals: 3.61–3.51 ppm belong to protons H3′ and H5′; the signal at 3.47 ppm indicates protons H2′ and H4′. Carbon C4′ forms a prochiral center [57]. The signals at 3.91 ppm and 3.72 ppm are from the protons of this center, H6′R and H6′S. The data for the aglycone moiety (4-methylsulfinylbutyl-) of GRN show the presence of a methylene chain (3.02–2.90 ppm; 2.82 ppm; and 1.98–1.78 ppm) attached to the thiohydroximate core, linking it to the sulfur atom. The signal at 2.71 ppm can be attributed to the terminal methyl group attached to the sulfur atom.

Similar to GRN, the anomeric proton in SBN is in the β-anomeric configuration (*J* = 9.4 Hz), but the signal for this 4.74 ppm peak is reduced due to its proximity to the suppressed water signal. A complex group of signals between 3.71 and 3.61 ppm and 3.49 and 3.25 ppm is characteristic of the glucose moiety. The multiplex at 3.71–3.63 ppm can be attributed to the methylene group of glucose (H6′R and H6′S). The aglycone part of SBN is well characterized by peaks at 7.28 ppm and 6.93 ppm, forming a doublet (*J* = 8.7 Hz), indicating the presence of a *p*-hydroxybenzyl group linked to a methylene group with a thiohydroximate core. The signal of the protons of these methylene groups has a value of 4.07 ppm (doublet). The resonance patterns for the β-thioglucoside part of GRN and SBN are different, probably due to the circular current effect that appears in the spectra of other GLSs with an aromatic structure, including glucotropaeolin or glucobrassicin [56]. The proton NMR spectrum of GRN and SBN is shown in Figure S2 (Supplementary Material).

MALDI-TOF analysis of purified GLSs from hoary cress leaves revealed two major compounds, GRN at *m*/*z* 437.1, and SBN at *m*/*z* 425.1. To confirm the identity of both GLSs, the parent peaks were further fragmented into characteristic ions (using MS/MS in negative ionization mode, Figure S3, Supplementary Material). The MS/MS spectrum of GRN showed a dominant peak at *m*/*z* 374 (mass range from 367.5 to 374.3 *m*/*z*), attributed to the GRN fragment formed due to the loss of the methylsulfinyl (CH_3_-SO-) group [58]. Additional characteristic peaks, representing sulfate and/or hydrogen sulfate ions, were observed in the mass range from 95.5 to 97.5 *m*/*z* [59]. Peaks in the mass range from 192.3 to 194.2 *m*/*z*, from 253.7 to 257.5 *m*/*z*, and from 271.4 to 273.3 *m*/*z* can be attributed to 1-thioglucose fragment and related fragments such as glucose-1-sulfate and 1-thioglucose-2-sulfate [58,60]. Deglucosylated fragments of GRN, especially the residues of the aglycon part, form peaks in the mass range *m*/*z* 237.8–241.1. Finally, the peak at *m*/*z* 356.5 corresponds to the desulfated fragment of GRN [61]. The mass spectrum of SBN shows, similarly to GRN, a dominant peak for the 1-thioglucose fragment (in the mass range from 192.4 to 194.4 *m*/*z*), together with its related fragments (glucose-1-sulfate and 1-thioglucose-2-sulfate). Moreover, the mass range *m*/*z* 95.1–97.0 indicates sulfate and bisulfate ions. The spectrum shows peaks corresponding to fragments related to the aglycone moiety, dominant peaks in the mass range from 179.5 to 181.5 *m*/*z* and from 228.1 to 230.1 *m*/*z* (related to desulfated and/or deglucosylated fragments of SBN), together with minor peaks at *m*/*z* 106.4 (4-methylphenol fragment) and 136.4 (4-iminoethylphenol fragment) [61,62]. Desulfated SBN fragments are represented in the spectrum as peaks at *m*/*z* 328.0 and 345.6 [61].

### 2.3. Transformation of Purified GRN to SFN by Myr from Garden Cress

To release the aglycone from GRN, we used a crude preparation enriched with Myr from garden cress seeds obtained by precipitation of the seed homogenate with ammonium sulfate [63]. This preparation showed an activity of 0.2 ± 0.02 μmol/(min × mg). Myr can be further purified practically to electrophoretic homogeneity by affinity chromatography with immobilized SFN [63]. However, for the effective release of SFN from GRN, such a highly pure preparation is not necessary, and the crude preparation obtained by precipitation with ammonium sulfate was sufficient.

**Figure 3:**
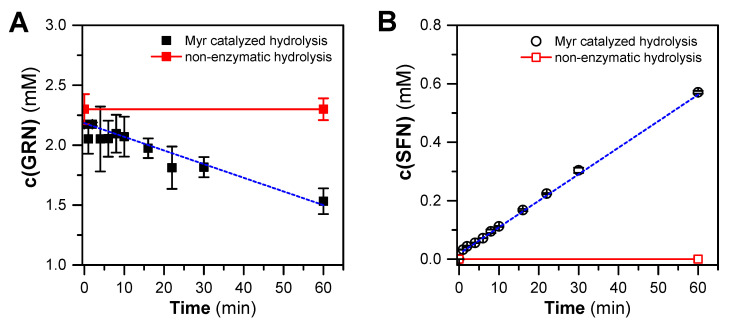
Time course of hydrolysis of GRN (purified from hoary cress) catalyzed by crude Myr preparation from garden cress monitored as a decrease in GRN content (**A**) and simultaneous formation of SFN (**B**) at 37 °C for 60 min. The composition of the reaction mixtures was as follows: 300 mM MES-NaOH buffer, pH 6.5, 0.1 mM L-ascorbic acid, 0.3 mg/mL proteins containing active Myr, and 2 mM GRN. Aliquots of the reaction mixture were taken at respective time intervals, and reaction in aliquots was stopped by addition of cooled MeOH (in a ratio of 1:2, −20 °C). The concentration of GRN and SFN was estimated by HILIC-based HPLC (see Section 4.5) and C18-RP-HPLC (see Section 4.9) analysis, respectively. The time course of GRN consumption or SFN formation yields linear dependencies that, by linear regression, result in correlation coefficients of r = −0.975 and r = 0.997 for 8 degrees of freedom. The straight-line slopes were −0.0114 and 0.00998 mM/min for GRN consumption and SFN formation, respectively.

In order to assess whether GRN purified from hoary cress is a suitable substrate for Myr from garden cress, GRN was incubated with crude Myr preparation for 0–60 min. After these time intervals, GRN and SFN concentrations were determined by HILIC-based HPLC and C18-RP-HPLC, respectively. From the kinetic course of GRN hydrolysis (Figure 3), it is clear that Myr from garden cress is capable of cleaving purified GRN with a reaction rate of 0.032 ± 0.011 μmol GRN/(min × mg of proteins). At the same time, we confirmed the SFN arising from GRN at a similar rate of 0.0316 ± 0.0011 μmol SFN/(min × mg of proteins), which indicates the fact that SFN is formed from consumed GRN in an equimolar ratio of about one. It could be concluded that only SFN is formed from GRN hydrolysis under our reaction conditions. Both the decrease in the GRN concentration and the increase in the SFN concentration appeared to follow a linear relationship, although some fluctuations were observed (Figure 3). This indicates that both GRN splitting and SFN origination followed a 0-order reaction, which is typical for simple enzyme reactions with such an excess of a substrate that the reaction is not limited by it during the entire time course.

**Scheme 1:**
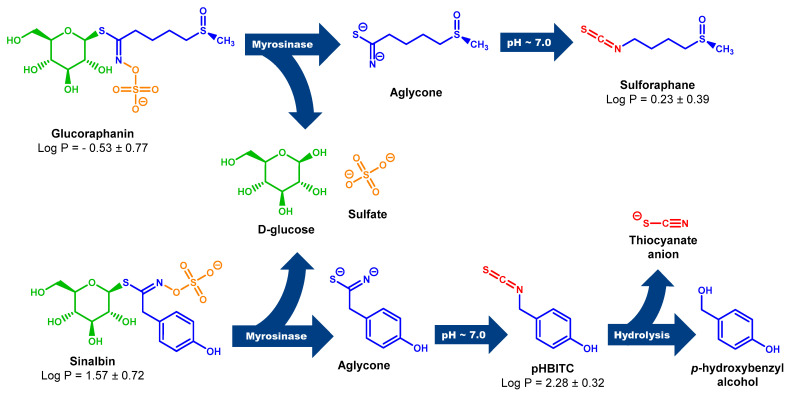
Myr cleavage of GRN and SBN with subsequent rearrangement of unstable aglycones to the corresponding ITCs. Unstable aglycones are formed from both GRN and SBN in the Myr reaction with the simultaneous release of D-glucose and sulfate anion [18,85]. At neutral pH, aglycones undergo rearrangement to the corresponding ITCs (SFN and pHBITC). Unlike SFN, which is relatively stable, pHBITC hydrolyzses to *p*-hydroxybenzyl alcohol, releasing the thiocyanate anion [18,85]. The log P (logarithm of partition coefficient in a two-phase mixture formed by water and n-octanol) was calculated using ACD/ChemSketch for academic and personal use (Advanced Chemistry Development, Inc., Toronto, ON, Canada).

Myr from garden cress is also able to cleave SBN (Figure S4 in Supplementary Material), but only half the amount of *p*-hydroxybenzyl-ITC (pHBITC) in relation to consumed SBN was detected. This may be related to the instability of pHBITC in the used reaction mixture. pHIBITC may decompose into *p*-hydroxybenzyl alcohol with the simultaneous release of a thiocyanate anion (Scheme 1). Such hydrolysis of benzyl-type ITC, especially at elevated temperatures, has been studied in detail [64].

### 2.4. Antimicrobial Properties of ITCs Derived from GLSs by Myr Reaction

**Figure 4:**
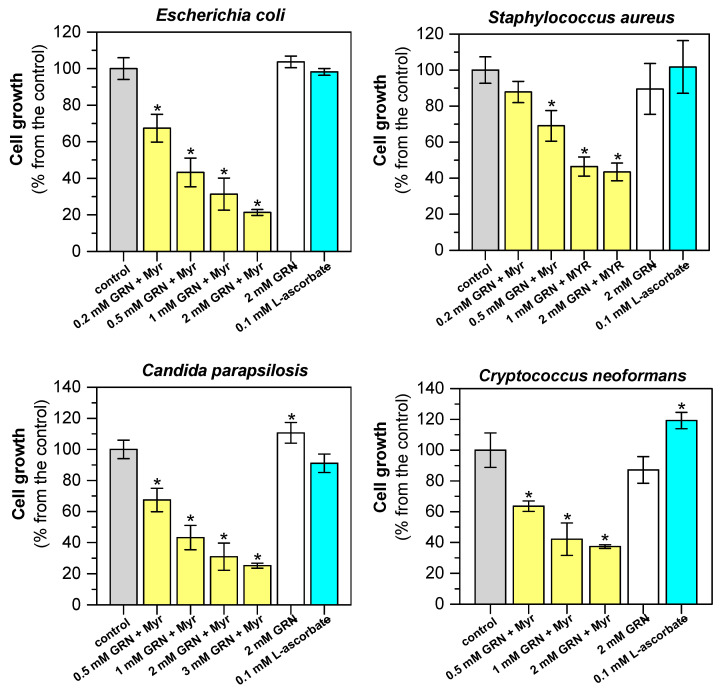
Antimicrobial effect of SFN formed from GRN by Myr on selected bacterial and yeast microbial models. Antimicrobial activity in vitro was evaluated after 12 h of growth of *E. coli* (CCM 3954), *S. aureus* (CCM 3953), *Cr. neoformans* (CCM 8312), or 24 h of growth of *Ca*. *parapsilosis* (CCM 8260) that were treated with SFN freshly generated by Myr-catalyzed GRN hydrolysis. Enzymatic hydrolysis of GRN was performed in a reaction mixture containing 300 mM MES-NaOH buffer, pH 6.5, with 0.1 mM L-ascorbic acid and 0.3 mg/mL proteins of the Myr crude preparation. The reaction was started by adding different initial concentrations of GRN (0.2–3.0 mM). Hydrolysis took place at 37 °C for 40 min before addition to the growth medium with the inoculum of the microbial cultures. The effects of GRN alone and L-ascorbic acid were also tested. In the control experiment, 300 mM MES-NaOH buffer with Myr was used. Statistical significance of differences between control samples and samples treated with SFN released from GRN by the Myr reaction, as well as with GRN or L-ascorbate alone, was calculated using a two-sample *t*-test, and asterisks indicate significant differences * *p* < 0.05.

The cytotoxic activity of ITCs released from GLS was evaluated in both prokaryotic and eukaryotic microbial models. *Escherichia coli* (CCM 3954) and *Staphylococcus aureus* (CCM 3953) have been selected as models of major bacterial pathogens causing infections with healthcare implications, particularly in elderly or immunocompromised patients [65]. *Cryptococcus neoformans* (CCM 8312) and *Candida parapsilosis* (CCM 8260) were selected as representatives of two genera of human opportunistic eukaryotic pathogens [66] for antifungal activity testing. Cytotoxicity assays were performed using the broth microdilution method under premixed conditions. In the premix method, the production of SFN from GRN was carried out by a reaction catalyzed with Myr; aliquots were added to the growth medium with the inoculum of model microorganisms. Figure 4 shows the effect of SFN produced by enzymatic hydrolysis of GRN on the growth of microorganisms. As expected, the growth inhibition of all tested microorganisms was proven, depending on the initial concentration of GRN. By increasing the initial concentration of GRN, more SFN is produced, resulting in a higher degree of growth inhibition. Non-hydrolyzed GLSs, L-ascorbic acid (a non-competitive activator of Myr [67]), MES-NaOH buffer, and crude Myr preparation did not inhibit the growth of microbial cells. On the contrary, the presence of L-ascorbic acid slightly but significantly increased the growth of *Cr. Neoformans,* and GRN slightly but significantly increased the growth of *Ca. parapsilosis* (Figure 4). A similar effect was observed when the used bacterial strains were exposed to in situ-generated SFN (Figure S6, Supplementary Material). The GRN HPs were generated simultaneously with the bacterial growth and resulted in complete inhibition of both bacterial strains.

The IC_50_ values (median inhibitory concentrations of SFN, MERCK s.r.o. Bratislava, Slovakia) when inhibiting the growth of model microorganism cells are shown in Table 3, which documents the effectiveness from 80 to 250 μM depending on the type of microorganism.

In an analogous experiment, but using SBN isolated from hoary cress as the second GLS, we did not observe significant effects on the growth of model microorganism cells.

## 3. Discussion

A key determinant of the effect of ITCs is the bioavailability of their precursors, which is influenced by several factors: the presence and amount of GLSs in the plant material, Myr stability, the degree of hydrolysis during the storage and/or processing of the plant material, the stability and physicochemical properties of GLSs/ITCs, and last but not least, the digestion and absorption of GLSs/ITCs in the human gastrointestinal tract [68,69,70]. Moreover, in vitro experiments showed that in the stomach acidic environment,, Myr is denatured, and 60–70% of GLSs are degraded [71]. Although the cleavage of GLSs also takes place in the digestive tract as a result of intestinal microbiota action, the degree of conversion of GLSs to ITCs is low since many bacteria from the genera *Lactobacillus*, *Lactococcus*, and *Enterobacter* [72] or the genus *Bifidobacterium* [73] form preferentially nitriles instead of ITCs, which are less reactive and, moreover, less effective. In addition, monitoring the effects and bioavailability of SFN and its precursors showed that by consuming a normal amount of cabbage vegetables, it is not possible to reach levels of ITCs in the tissues that would have anti-carcinogenic effects [74]. These observations indicate that to achieve an effective concentration of ITCs in cells, the creation of functional preparations enriched with GLSs and Myr is preferable and needs to be developed. For the activation of GLSs alone by Myr, the following three options are offered: (1) in vivo activation, when hydrolysis of GLSs and formation of ITCs occurs directly in the gastrointestinal tract, but the control of the reaction conditions (e.g., pH) is questionable; (2) ex vivo activation, when the components (GLSs and Myr) would be mixed prior to ingestion, where the reaction conditions might be more controllable; and (3) ex vivo activation of GLSs by bacterial thioglucosidases or bacterial cells. However, with ex vivo activation, the rate of absorption of the formed ITCs in the gastrointestinal tract is automatically associated. An alternative option is to target the production of foods enriched in GLSs (from natural sources or synthetically prepared) and consume them with a Myr preparation that would act only in the intestinal tract, where nutrient absorption occurs to the greatest extent.

To obtain an appropriate amount of free SFN, it is necessary to isolate both GRN and the active preparation, Myr. The most common source of GRN is broccoli (*Brassica oleracea* var. *italica*), the amount of which varies between varieties/cultivars [75]. Various strategies for GRN isolation and purification have been improved using different techniques, e.g., solid phase extraction combined with preparative HPLC [76] or high-speed counter-current chromatography [25]. These methods usually require complex chromatographic systems and are rather more expensive techniques compared to traditional chromatography methods. Although the final products are highly pure, the efficiency of these processes is usually lower [25].

Recent studies of various *Brassica* plants have revealed hoary cress characterized by allelopathic [77,78] and antioxidant properties [79,80]. This plant contains, in addition to GRN, only a small number of other GLSs [81,82,83,84], which makes it a preferred source of GRN. Moreover, the GLS profile from our point of view revealed not just remarkably high values of GRN content, but in addition, it revealed that the profile is absolutely dominated by GRN and SBN (for chemical structures, see Scheme 1).

Starting with hoary cress as a source of GRN, we proposed the following three-step laboratory isolation procedure: extraction of total GLSs from plant material and removal of plant pigments (1st phase); capture and enrichment of GLSs using IEC on DEAE-Sephadex A-25 (2nd phase); and GRN purification using GPC on Sephadex LH-20 (3rd phase). The decolorization of the extract prepared from the plant material using activated charcoal proved to be a necessary step before chromatographic purification. This step proved to be suitable for preventing the degradation of the Sephadex-based matrixes by their irreversible adsorption, which is usually absent in protocols for GLS isolation. After the extraction of total GLSs, we further purified GLSs by chromatography, first by IEC on DEAE-Sephadex A-25 and then by GPC on Sephadex LH-20. IEC was mainly used to trap anions (GLSs due to the sulfate part) from plant extracts from different plant parts. The possibility of capturing GLSs on DEAE-Sephadex A-25 has been known for a long time (see, e.g., [86]). GRN represented 11.0 and 12.6% in leaves and flowers, respectively, of the total amount of metabolites. However, SBN was represented higher in leaves by 5.6% than in flowers, where it represented only 3.0% (Table 1). IEC is the most widely used method of capturing GLSs, and in combination with desulfation directly on the column with an appropriately selected desulfatase and subsequent leaching of anion-free GLS residues, it is also used for the preparation of desulfated GLSs [87]. However, the natural GLSs contain a sulfate moiety, and therefore, we avoided the desulfatation step and worked with them in intact form.

The purity of GRN was greatly increased to 96% by the GPC Sephadex LH-20 column, which allowed us to separate GRN from other substances, particularly SBN, as the second major GLS of hoary cress (Figure 2). As a by-product, we obtained SBN with a purity of 92% [88]. An alternative method for the isolation and purification of GRN was described by Powell et al. [83]. Their protocol is based on the batch extraction of GRN from hoary cress under defined conditions that allow a GRN yield of up to 30 mg GRN/gram of dried hoary cress, but there is no mention of the purity of the isolated GRN. Although our GRN yield was 22.9 ± 1.2 mg GRN/g dry plant material, the purity level was estimated to be 96%. The structure of both obtained GLSs was later proven by ^1^H-NMR spectroscopy and MS/MS analysis (Table 2, Figures S2 and S3 in Supplementary Material).

The essential task was to find out if an appropriate amount of free SFN is generated during the enzymatic cleavage of GRN, which could exhibit any biological effects. We subjected the isolated GRN to enzyme hydrolysis with a crude Myr preparation obtained from swollen garden cress seeds by isoelectric precipitation with ammonium sulfate [63]. Although Myr preparation is not an electrophoretically homogeneous enzyme, it has a few advantages against purified enzymes [63], such as easy and fast preparation with a high yield and better storage stability at 4 °C (Figure S5, Supplementary Material). The catalytic ability of crude Myr preparation to decompose GRN and form SFN was confirmed by an RP-HPLC analysis. We observed almost a 100% conversion of GRN to SFN under the used experimental conditions (Figure 3) at a reaction rate comparable to that measured with a purified enzyme [63,89,90]. Based on this observation, it can be concluded that the isolated GRN and the crude Myr preparation represent a suitable GLS-Myr system for the generation of SFN in biological samples under in situ conditions.

To verify the antibacterial and antifungal effects of SFN generated from GRN by enzyme transformation, we tested the cytotoxicity of HPs using a premix approach after their addition to the medium for the growth of model microbial cells (Figure 4). The chosen microbial strains were standard reference strains used for antibacterial or antifungal susceptibility tests, respectively. The antimicrobial effect of commercially available SFN is achieved at concentrations close to 100 μM (Table 3). Myr, L-ascorbic acid, or GRN alone do not show any antimicrobial effects. However, if they are blended together under premix conditions, we observed an inhibition of the growth of all model organisms in a manner dependent on the concentration of GRN in the mixture (Figure 4). An inhibition effect was observed too when SFN was generated under in situ conditions, i.e., adding the reaction mixture to the inoculum without any pre-incubation (Figure S6, Supplementary Material). ITCs can have more than one target in cells, and the mechanism of their antimicrobial activity is species-specific. Notably, SFN has been reported to have diverse targets in eukaryotic cells and modulate numerous cell signaling pathways [91], some of which are specific to a particular biological model [92]. The present study is an attempt to prepare a suitable preparation of GRN and Myr for possible application. It was not intended to reveal the detailed mechanism of action of SFN on model microorganisms. This will require further research, and it is likely that the effects will show features of specificity depending on the microorganism being studied.

The physicochemical and reactivity properties of ITCs are well documented [20]. Since they are reactive electrophiles, they can react with the -SH group to form the corresponding dithiocarbamates of small molecules (such as dihydrolipoic acid or glutathione) of proteins (such as thioredoxin, SH proteases, several dehydrogenases, etc.) and thus induce effects on redox balance, metabolic processes, and various signal transduction pathways. They can also react with -NH_2_ groups of proteins to form N,N-disubstituted thioureas and potentially inhibit enzymes, disrupt proton bridges stabilizing protein secondary structure, block protein ubiquitination, etc., thus altering the course of both metabolic and signal transduction pathways [93,94,95]. In addition to the chemical properties and structure of the ITCs, the components of the growth medium also influence the effect of the ITCs. Andini et al. [96] showed that by reducing the concentration of nucleophiles in the growth medium, the antimicrobial activity of ITCs was significantly improved. The interaction of ITCs with cells, especially with the cell surface, is decisive, i.e., the cell membrane of animal cells or the cell wall and plasma membrane in microorganisms. Both Gram-positive and Gram-negative bacteria have a cell wall formed by peptidoglycans, under which is a phospholipid bilayer of the plasma membrane with integral membrane proteins. Gram-negative bacteria, however, have a unique outer membrane covering the cell wall. The outer membrane consists of phospholipids, lipopolysaccharides, integral membrane proteins, and lipoproteins and is usually attributed to a higher resistance to lysozyme attack and the action of antibiotics [97], as well as to various ITCs [24]. We also observed a more pronounced effect of SFN on Gram-positive *S. aureus* than on Gram-negative *E. coli* (Table 3).

Although cell surface barriers are well permeable to low-molecular-weight (phyto)chemicals with lipophilic properties, SFN and GRN may be an exception due to their more polar nature. The effectiveness of ITCs can also be influenced by the composition of the cell surface and the ability of their components to react with the -NCS group of ITCs. For example, treatment of the RAW264.7 macrophage cell line with benzyl isothiocyanate (hydrophobic ITC) resulted in its covalent binding to phosphatidylethanolamine (PE) [98]. PE is the dominant glycerophospholipid of the plasma membrane of Gram-negative bacteria [99]. Subsequently, there is a possibility that even lipophilic ITCs can react with PE in the membranes of Gram-negative bacteria, which can lead to their accumulation in the membranes and various subsequent reactions. On the other hand, this reaction could prevent the entry of even lipophilic ITCs into the intracellular space. In eukaryotic microorganisms, we found that *Cr. neoformans* was slightly more sensitive to SFN than Ca. *albicans*. The reason for this fact remains unknown, but a similar behavior was observed when treating these yeasts with other ITCs [24]. Since yeasts are eukaryotic cells, there could be few similarities with animal cells in the efficacy of ITCs, excluding those based on interactions with cell wall structures. Therefore, the inhibitory effect of ITCs on yeast cells is thought to have some similar features to that of mammalian cells. However, SFN shows an effect on microbial eukaryotes at 0.1 mM (Table 3) and on mammalian cells at ten times lower concentrations [42]. Recently, the effect of enzymatically generated SFN on murine leukemia cells was studied by means of recombinant *Arabidopsis thaliana* Myr [39], which was prepared and characterized by the group of Dr. Rebroš [89,90]. This study showed that the mixture of GRN with recombinant Myr in the presence of 10 μM L-ascorbic acid is able to produce the sufficient level of SFN to be cytotoxic against L1210 cells and induce cell death via autophagy [39,42].

Although the obtained results only represent the first step in the characterization of the mechanism of the enzyme preparation of the active form of SFN, capable of suppressing the growth of prokaryotic and eukaryotic cells, they provide a starting point for further research and development of functional foods with cancer-preventive properties.

## 4. Materials and Methods

### 4.1. Chemicals and Plant Materials

#### 4.1.1. Chemicals

D,L-SFN (1-isothiocyanato-4-(methylsulfinyl)-butane) and the potassium salt of its GLS precursor GRN (4-methylsulfinylbutylglucosinolate), DEAE-Sephadex A-25 and Sephadex LH-20, formic acid, ammonia, acetonitrile, sinigrin, L-ascorbic acid, MES, BSA, Mueller Hinton Broth No. 2, 9-aminoacridine, AMP, ATP, glucose-6-phosphate, acetyl-CoA, TFA were all from Sigma-Aldrich and supplied via MERCK spol. S.r.o., (Bratislava, Slovakia); peptone for bacteriology, agar (Biolife Italiana, Milano, Italy). Unless otherwise noted in the text, all chemicals were from MERCK and were of analytical grade.

#### 4.1.2. Plant Materials

GRN and SBN were isolated from hoary cress (*Lepidium draba*) in 2019. Plant material of hoary cress was collected from its natural habitat during the flowering period (early May to June).

Myr was isolated from the swollen seeds of garden cress (*Lepidium sativum*) purchased commercially (Garden Seeds BV, Enkhuizen, The Netherlands).

### 4.2. Extraction of Total GLSs from Hoary Cress

After harvesting the hoary cress, whole plants were fried to a constant weight at 100 °C for 2 h. The leaves and flowers of the dry plant were separated and then ground in a coffee grinder (Tefal COFFEE GT110838, Rumilly, Haute-Savoie, France) for 1 min. GLS extraction from 30 g of dried plant material (leaves or flowers of hoary cress) was performed using 200 mL of 80% (*v*/*v*) MeOH according to Crocoll et al. [88] with some modifications. The extraction was carried out in an Erlenmeyer flask (500 mL) under continuous shaking at 200 rpm (orbital shaker, UNIMAX 2010, Heidolph, Germany) at room temperature for 2 h. After extraction, the mixture was filtered through a Büchner funnel with filter paper (pore size 12–15 μm, Boeco Hamburg, Germany), and the plant residues collected were subjected to a second extraction in 80% (*v*/*v*) MeOH under the same conditions. Both filtrates were combined, and plant pigments and other colored impurities were adsorbed with activated charcoal (Norit A, Serva, Heidelberg, Germany) as follows: 6 g of powdered activated charcoal was added to 400 mL of extract. Adsorption was carried out under continuous shaking at room temperature for 30 min at 200 rpm. After removing activated charcoal from the mixture by filtration, the colorless filtrate was first concentrated on a rotary evaporator at 40 °C (under reduced pressure), then filtered through a 0.45 μm PTFE syringe filter (Macherey-Nagel, Düren, Germany), and stored at −20 °C.

### 4.3. Isolation of GLS Anions Using Ion-Exchange Chromatography (IEC) of Plant Extracts

The possibility of purifying GLSs on anion chromatographic matrices has so far been rarely studied, but its applicability has been verified [100]. GLSs present in the methanolic plant extract were captured by ion-exchange chromatography (IEC) on a column (15.5 × 4.5 cm) packed with DEAE-Sephadex A-25 and equilibrated with 0.5 M CH_3_COOH-NaOH buffer (pH 5). Subsequently, 100 mL of the extract was applied to the column and washed first with a mixture of 380 mL of formic acid/isopropanol/water (3:2:5, *v*/*v*/*v*) and then with 400 mL of deionized H_2_O. GLSs captured on the column were then eluted with 680 mL of 0.5 M K_2_SO_4_ in 3% (*v*/*v*) isopropanol. Finally, the column was washed with deionized H_2_O and re-equilibrated with 0.5 M CH_3_COOH-NaOH buffer (pH 5) to prepare for further chromatography. The eluate was concentrated on a rotary evaporator to 100 mL (at 40 °C, under reduced pressure), and the formed K_2_SO_4_ crystals were removed by filtration through a frit funnel (porosity 90–160 µm). The remaining dissolved K_2_SO_4_ was removed by adding 200 mL of 96% ethanol to the filtrate. K_2_SO_4_ precipitates were removed by centrifugation for 15 min at 7100× *g* at 4 °C (Centrifuge 5430R, Eppendorf, Hamburg, Germany). The presence of GLSs and their quantifications were determined by HILIC (Section 4.5). The supernatant was concentrated by rotary evaporation, lyophilized, and stored at −20 °C until further use.

### 4.4. Purification of GRN in Samples Obtained by IEC

Individual GLSs (GRN and SBN) in samples obtained with IEC can be separated from each other and freed from other contaminants using two different chromatographic techniques: gel permeation chromatography (GPC) and thin-layer chromatography (TLC).

#### 4.4.1. Gel-Permeation Chromatography of GLSs

The possibility of GPC of GLSs using a methanol–water mobile phase was demonstrated [101]. The column (109 × 1.5 cm) was packed with epichlorohydrin cross-linked dextran (which formed chlorohydroxypropyl linkages), Sephadex LH-20, and equilibrated with 80% (*v*/*v*) MeOH. GLS samples (obtained after IEC) were dissolved in 80% (*v*/*v*) MeOH to a final concentration of 100 mg/mL, and 4 mL aliquots of the sample were applied to the column. GLS were eluted under isocratic conditions with 80% (*v*/*v*) MeOH at a flow rate of 1 mL/min. Fractions of 2 mL were collected, and the presence of GLSs was determined by HILIC (Section 4.5). Fractions containing particular GLSs were pooled and lyophilized after removing the organic solvent by rotary evaporation. Purified GLSs were stored at −20 °C until further use.

#### 4.4.2. Thin-Layer Chromatography (TLC) of GLSs

GLS purification by TLC was performed on pre-coated silica gel 60 F_254_ plates (20 × 20 cm, Sigma Aldrich) cut to 10 × 10 cm plates. The developing solvent was a mixture of isobutanol/*n*-propanol/CH_3_COOH/H_2_O (3:1:1:2, *v*/*v*/*v*/*v*), as described by Matsuo [102]. Plates were activated at 100 °C for 1 h before use. GLSs (up to 2 mg/plate) dissolved in 80% (*v*/*v*) MeOH were deposited gradually in the form of a thin line in several layers on the surface of the TLC plate (there were 2 separate TLC plates). After the separation was complete, the spots were visualized with UV light (both plates at 312 nm) or with iodine vapors (one plate). Detected spots were scraped from silica gel (only from the UV–visualized plate) and extracted with 80% (*v*/*v*) MeOH with intensive vortexing for 5 min. Insoluble particles were removed by centrifugation at 12,000× *g* for 1 min at room temperature (MiniSpin, Eppendorf, Hamburg, Germany), the supernatant was evaporated using a vacuum concentrator (Speed-Vac, Eppendorf, Hamburg, Germany), and the samples were used for analysis by HILIC (Section 4.5) and ^1^H-NMR spectroscopy (Section 4.6).

### 4.5. Detection and Quantification of GLSs Using HILIC

Qualitative and quantitative detection of GLSs in samples after IEC, GPC, and TLC was performed using an isocratic HPLC system. This system consisted of an LC 10ATvp pump and UV–VIS detector from Shimadzu Corporation (Kyoto, Japan) and a SpectraSYSTEM AS3000 autosampler from Thermo Separation Products (Piscataway, NJ, USA), as well as a HILIC column containing sulfoalkylbetaine zwitterionic functional groups (150 × 4.6 mm, 5 μm, EC 150/4.6 Nucleodur HILIC 5 μm, Macherey-Nagel, Düren, Germany). HPLC measurements were performed according to the procedure of Wade et al. [103]. After injection of 20 μL of sample, GLSs were isocratically eluted with 15 mM ammonium formate in 70% (*v*/*v*) acetonitrile at pH 5 and at a flow rate of 0.5 mL/min and detected at 229 nm in a UV detector. Chromatograms were evaluated with Clarity Lite™ 2.1 software (DataApex, Prague, Czech Republic). The purity of the final preparations was controlled by an additional run on HILIC and was calculated as the ratio of the peak area of individual GLSs to the total area of the peaks detected in the chromatogram at 229 nm. The amount of individual GLSs was calculated from the peak areas at 229 nm relative to the peak of the internal standard (potassium sorbate) using the relative response factor (evaluated in our laboratory).

### 4.6. Verification of the Structure of GLSs with ^1^H-NMR

^1^H-NMR spectra were measured on the 600 MHz NMR spectrometer (Varian, Palo Alto, CA, USA) with the VNMRS console and gHX nanoprobe. Standard pulse sequences from the VnmrJ 4.2 library were used. ^1^H spectra were measured at 1500 Hz speed using tnnoesy sequence with 1 s of relaxation time with water presaturation, 100 ms of mixing time, and 4 s of acquisition time. A minimum of 256 repetitions/frames were accumulated during one measurement. The spectra were analyzed with MestreNova 12.0.1 (Mestrelab Research S.L, Santiago, Spain) using automatic baseline correction. Spectra were referenced to the TSP signal (0 ppm).

### 4.7. Identification of GLSs by MALDI-TOF Mass Spectrometry

GLS was dissolved in 50% (*v*/*v*) MeOH to a concentration of 10 mg/mL and was applied to the MALDI stainless steel target together with 0.5 μL of 9-aminoacridine (10 mg/mL in a 1:1 mix of methanol and 0.1% (*v*/*v*) TFA, Fluka, Gillingham, Dorset, UK). The spots were allowed to dry and then analyzed in negative ion mode. Analysis was performed by MALDI-TOF/TOF mass spectrometry in the range (*m*/*z*) from 70 to 700 Da on AUTOFLEX III Smartbeam MALDI-TOF/TOF (Bruker Daltonics, Bremen, Germany). External calibration was performed using citrate (M-H (monoisotopic) 192.03 Da), glucose-6-phosphate (M-H (monoisotopic) 259.01 Da), AMP (M-H (monoisotopic) 391.19 Da), ATP (M-H (monoisotopic) 505.99 Da), and acetyl-CoA (M-H (monoisotopic) 505.99 Da) as calibrants.

### 4.8. Crude Myr Preparation from Garden Cress and Measurement of Its Activity

Myr, serving to activate GRN, was obtained from garden cress seeds by isoelectric ammonium sulfate fractionation, as reported previously by Galadova et al. [63]. The freshly prepared Myr was stored at 4 °C. The concentration of proteins was routinely determined by the method of Bradford [104] with bovine serum albumin (BSA) as the standard. The Myr activity of enzyme preparation was estimated spectrophotometrically by the detection of D-glucose released upon sinigrin hydrolysis. The amount of D-glucose was assayed by the glucose–oxidase–peroxidase-coupled enzyme method, as described elsewhere [63]. The transformation of GRN on SFN by Myr reaction was assessed by direct measurement of both GRN decreases and SFN increases by HILIC (Section 4.5) and C18-RP-HPLC (Section 4.9), respectively.

### 4.9. Qualitative and Quantitative Analysis of SFN by C18-RP-HPLC

The decrease in GRN and SFN formation after Myr hydrolysis of GRN was determined by HILIC and RP-HPLC. Enzymatic hydrolysis of GRN was performed in 300 mM MES-NaOH buffer (pH 6.5) with 0.1 mM L-ascorbic acid and 0.3 mg/mL of the enzyme fraction from garden cress seeds. The reaction was initiated by the addition of 3 mM GRN (substrate), and the reaction was allowed to proceed at 37 °C for 60 min. Aliquots of the reaction mixture were taken at different time intervals to monitor the reaction, and the reaction in these aliquots was stopped by the addition of precooled MeOH (−20 °C in a 1:2 ratio). Precipitated proteins were removed by filtration through 0.22 μm PTFE syringe filters, and the filtrates were used to monitor either SFN formation or GRN decrease. The reaction mixture, to which precooled MeOH was first added, and then the substrate, served as a blank. In parallel, the possibility of non-enzymatic hydrolysis of GLS in the reaction mixture without enzyme was monitored. The assays were performed in duplicate, and the results are expressed as mean ± SD.

The amount of GRN in the obtained samples was determined using the HILIC procedure described in Section 4.5. The amount of SRN was determined by C18-RP-HPLC on a Shimadzu HPLC system, (described in Section 4.5) with an C18 RP column (250 × 4 mm, 5 μm, EC 250/4 Nucleodur 100–5 C18ec column, (Macherey-Nagel). For analysis, 20 μL of sample was injected into the column and eluted isocratically with 50% (*v*/*v*) acetonitrile containing 0.1% (*v*/*v*) H_3_PO_4_ at a flow rate of 0.5 mL/min (according to No. 077 protocol available on the internet for isothiocyanates analysis [105]). Commercially available SFN was used as the standard.

### 4.10. Quantitative Analysis of Thiocyanate Anions

The hydrolysis of SBN was monitored as the formation of thiocyanate anions (SCN^−^) by a modified method, according to Hovinen et al. [106]. The reaction mixture was prepared as described in Section 4.9, except that GRN was replaced by SBN. The aliquots of reaction mixture (after removing the precipitated proteins) were mixed with Fe^3+^ reagent (200 mM FeCl_3_ in 1 M HCl) in a ratio of 2:1 (*v*/*v*). The concentration of KSCN in the reaction mixture after hydrolysis was estimated spectrophotometrically by measuring the absorbance at 440 nm. The decrease of SBN was monitored by HILIC-dependent HPLC (Section 4.5).

### 4.11. Antimicrobial Assay

Testing of the antimicrobial effects of HPs obtained by cleavage of GRN by Myr was performed using the following microbial strains obtained from the Czech collection of microorganisms (Masaryk University, Brno, Czech Republic). Prokaryotes: *Escherichia coli* (Gram-negative bacteria, clinical isolate, CCM 3954, ATCC 25922) and *Staphylococcus aureus* (Gram-positive bacteria, clinical isolate, CCM 3953, ATCC 25923) as reference strains for antibacterial susceptibility testing; Eukaryotes: *Cryptococcus neoformans* (encapsulated yeast from division *Basidiomycota*, class *Tremellomycetes*, CCM 8312, ATCC 90112) and *Candida parapsilosis* (yeast from division *Ascomycota*, class *Saccharomycetes*, CCM 8260, ATCC 22019) as reference strains for antifungal susceptibility testing. Bacterial strains were cultured on Mueller–Hinton agar plates [107], yeast strains on Sabouraud glucose agar plates (with the following composition of 20 g/L D-glucose, 1 g/L peptone, and 2% (*w*/*v*) agar, pH adjusted to 5.5).

The starter inoculum of the tested strains was obtained by submerged cultivation (on an orbital shaker at a shaking frequency of 4 Hz) in 10 mL of a specific growth medium (without agar) at 37 °C (bacterial strains) or 27 °C (yeast strains) for 16 h. Subsequently, the inoculum was diluted in 3-fold concentrated growth medium to reach a final organism density of 1 × 10^6^ cells/mL (test culture) and then used to evaluate the antimicrobial activity of the HPs obtained by the Myr reaction.

The effect of HPs from GRN on microbial strains was tested by the microdilution method under pre-mixing conditions (premix), which allows first mixing GRN and Myr and then applying the HPs formed from GRN to the microbial culture under test. Another method featured the generation of GRN HPs in situ, when the GRN and Myr were directly added to the microbial culture under test. This method was applied only to monitoring the effect on bacterial strains. Myr-catalyzed hydrolysis of GRN was carried out in 120 μL of 300 mM MES-NaOH buffer, pH 6.5, containing 0.1 mM L-ascorbic acid and 0.3 mg/mL crude Myr preparation. The reaction was initiated by the addition of GRN. The final concentration of GRN in the reaction mixture was in the range of 0 to 3 mM. After 40 min of incubation at 37 °C, the reaction mixture was mixed with 60 μL of test culture (prepared as described above) and incubated under continuous shaking conditions at 37 °C (bacterial strains) or 30 °C (yeast strains) for an additional 12 h (for *E. coli*, *S. aureus*, and *Cr. neoformans*) or 24 h (for *Ca. parapsilosis*), respectively. Cell growth was monitored spectrophotometrically at 630 nm using a 96-well microtiter plate reader (ELISA reader ELx808, BioTek, Winooski, VT, USA). Each assay was performed in tetraplicates, and the data were expressed as mean ± SD.

Commercially available SFN was used to determine the median inhibitory growth concentration of SFN (IC_50_) per individual microbial strain, and IC_50_ values were determined from the toxicity curves (growth dependence of each strain on SFN concentration) by nonlinear regression analysis using OriginPro 8.5 software (OriginLab Corporation, Northampton, MA, USA). Cell growth in the absence of SFN was used as a control.

### 4.12. Data Evaluation

Unless otherwise described, all values represent the mean ± SD from triplicates. A two-sample *t*-test was calculated by Origin 8.5 software. *p* < 0.05 was the limit of statistical significance.

## 5. Conclusions

For the isolation of GLSs, we designed a two-step chromatographic procedure based on the principles of ion exchange and gel permeation, which allowed us to obtain intact GRN and SBN from cruciferous cress weeds with purities of 96% and 92%, respectively. The presented procedure can be applied to the purification of other types of GLSs from various plant sources. However, by changing the plant material and types of GLSs, the procedure requires some optimalizations. Measurements of the thioglucosidase activity of Myr prepared from garden cress seeds against intact GLSs showed that both purified GLSs can serve as substrates and can be transformed into active ITCs. However, unlike SFN, which persists in the ITC form, pHBITC formed from SBN rapidly hydrolyzes to *p*-hydroxybenzyl alcohol, resulting in a loss of antimicrobial activity (Scheme 1). In contrast, HPs formed from GRN Myr reactions significantly inhibited the growth of model bacterial and yeast strains.

## 6. Patents

The method of isolation of GRN from hoary cress (*L. draba*) used in this work is registered at the Industrial Property Office of the Slovak Republic as utility model under the ref. No. SK 9579 Y1.

## Figures and Tables

**Table 1:** Yield of GRN and SBN obtained from hoary cress by IEC.

Plant Part Used for Isolation	Yield of Total Metabolites after IEC ^1^ mg/g (drm)	Glucoraphanin ^2^ mg (GRN)/g (drm)	Sinalbin ^3^ mg (SBN)/g (drm)
leaves	260.3 ± 18.8	28.7 ± 2.4	14.7 ± 0.5
flowers	266.6 ± 19.0	33.5 ± 3.5	7.9 ± 0.8

^1^ This value represents the amount of lyophilized product after IEC (obtained by weighing), which were isolated from one gram of dried plant material. ^2,3^ The content of GRN or SBN was determined by means of HILIC-based HPLC.

**Table 2:** ^1^H-NMR chemical shift assignments (ppm) of GLSs (600 MHz in D_2_O).

	Glucoraphanin		Sinalbin	
	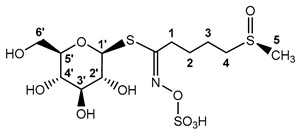		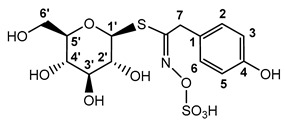	
H	Chemical Shift (δ (ppm))	Multiplicity (*J* (Hz))	Chemical Shift (δ (ppm))	Multiplicity (*J* (Hz))
Glucose moiety				
H1′	5.04	1H, d, *J* = 9.9 Hz	4.74	1H, d, *J* = 9.4 Hz,
H2′	3.47	2H, dd, *J* = 9.9, 9.0 Hz	3.38–3.30	2H, m
H3′	3.61–3.53	2H, m	3.38–3.30	2H, m
H4′	3.47	2H, dd, *J* = 9.9, 9.0 Hz	3.46–3.38	1H, m
H5′	3.61–3.53	2H, m	3.25	1H, ddd, *J* = 9.8, 4.5, 2.8 Hz
H6′*R*	3.91	1H, dd, *J* = 12.6, 2.2 Hz	3.71–3.63	2H, m
H6′*S*	3.72	1H, dd, *J* = 12.6, 5.9 Hz	3.71–3.63	2H, m
Aglycone moiety				
H1	2.82	2H, t, *J* = 7.6 Hz		
H2	1.98–1.78	4H, m	6.93	2H, d, *J* = 8.7 Hz
H3	1.98–1.78	4H, m	7.28	2H, d, *J* = 8.7 Hz
H4	3.02–2.90	2H, m		
H5	2.71	3H, s	7.28	2H, d, *J* = 8.7 Hz
H6			6.93	2H, d, *J* = 8.7 Hz
H7			4.07	2H, d, *J* = 3.8 Hz

s = singlet; d = doublet; t = triplet; dd = doublet of doublets; ddd = doublet of doublet of doublets; m = multiplet.

**Table 3:** Values of SFN IC50 for SFN estimated for model microorganisms.

Microorganism	IC_50_ for SFN (μM)
*Escherichia coli* (CCM 3954)	250.6
*Staphylococcus aureus* (CCM 3953)	109.8
*Candida parapsilosis* (CCM 8260)	137.0
*Cryptococcus neoformans* (CCM 8312)	82.7

Commercially available SFN was used in measurements.

## Data Availability

The data presented in this study are available on request from the corresponding author.

## Supplementary Materials

The following supporting information can be downloaded at https://www.mdpi.com/article/10.3390/plants13070995/s1, Figure S1: Chromatograms from preparative TLC of GLSs from hoary cress and ^1^H-NMR spectrum of major spots, Figure S2: ^1^H-NMR analysis of GRN and SBN obtained after complete purification, Figure S3: MALDI-TOF/TOF (MS/MS) spectrum of the parental peaks with *m*/*z* 437.1 Da (purified GRN) and 425.1 Da (purified SBN), Figure S4: Time course of hydrolysis of SBN (purified from hoary cress) catalyzed by crude Myr preparation from garden cress, Figure S5: Long-time Myr stability measurement, Figure S6: Effect of in situ generated SFN on growth of *E. coli* and *S. aureus*.

## Abbreviations

AMP	adenosine 5′-monophosphate
ATP	adenosine 5′-triphosphate
DEAE-	2-diethylaminoethyl-
drm	dry plant material
DTT	DL-dithiothreitol
GLS(s)	glucosinolate(s)
GPC	gel permeation chromatography
GRN	glucoraphanin
HILIC	hydrophilic interaction liquid chromatography
HP(s)	hydrolysis product(s)
IEC	ion-exchange chromatography
ITC(s)	isothiocyanate(s)
MES	2-(N-morpholino)ethanesulfonic acid
Myr	myrosinase
PE	phosphatidylethanolamine
pHBITC	*p*-hydroxybenzyl isothiocyanate
PTFE	polytetrafluoroethylene
SBN	sinalbin
SFN	sulforaphane
TFA	trifluoroacetic acid
