# Supplementary material for: The Antimicrobial Effects of Myrosinase Hydrolysis Products Derived from Glucosinolates Isolated from Lepidium draba
Source: Plants (Basel). 2024 Mar 30;13(7):995. doi: 10.3390/plants13070995 (PMC11013450; doi:10.3390/plants13070995)
Supplement: Supplementary file 1 [file plants-13-00995-s001.zip › plants-2872043-supplementary.pdf]

Supplementary material

The Antimicrobial Effects of Myrosinase Hydrolysis Products Derived from Glucosinolates Isolated from *Lepidium draba*

Zoltán Polozsányi et al.

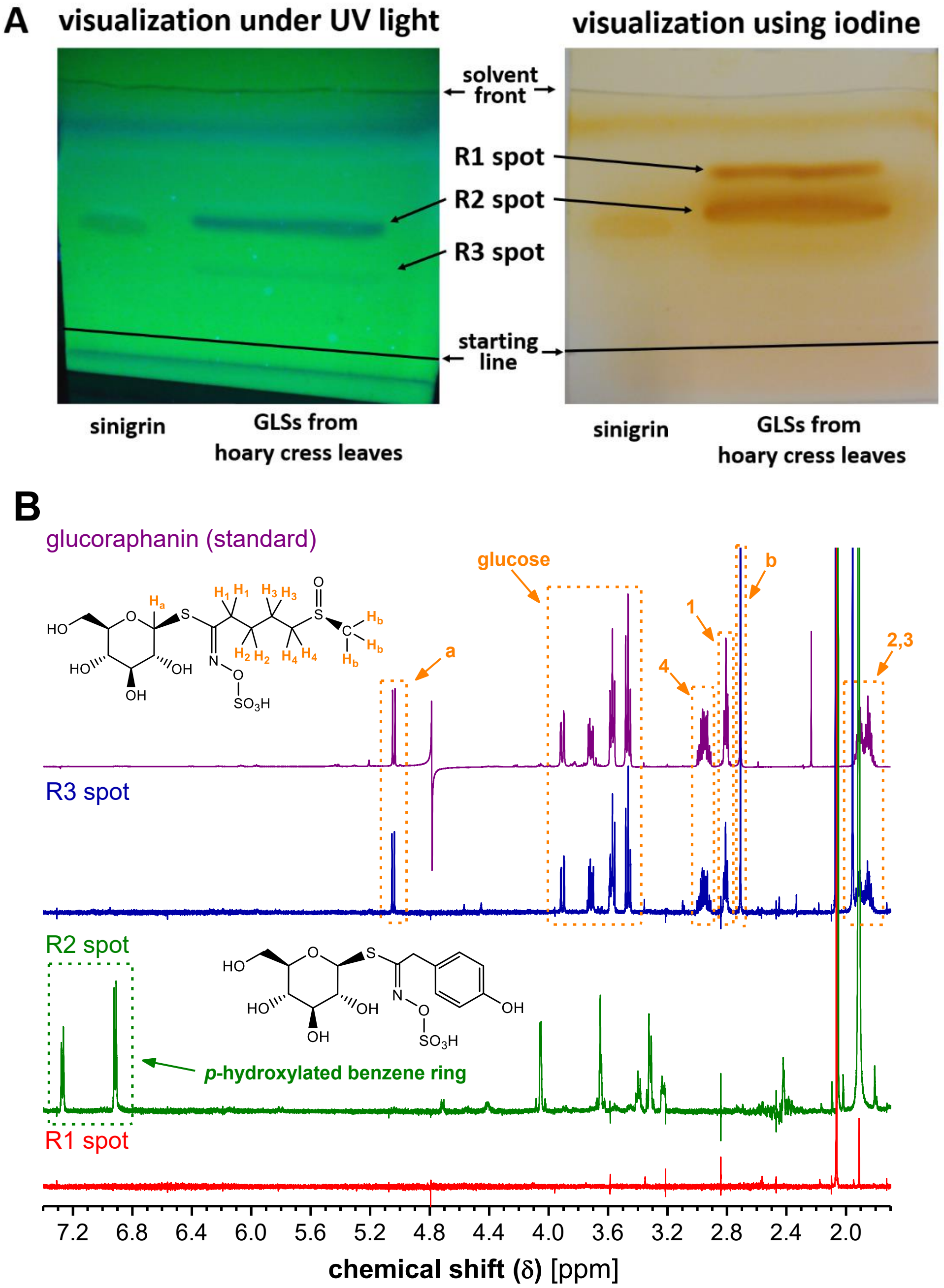

Figure S1. Chromatograms from preparative TLC of GLSs from hoary cress (A) and ^{1}H-NMR spectrum of major spots (B). The resolved spots R1 (R_f = 0.705), R2 (R_f = 0.544) and R3 (R_f = 0.367) were analyzed by HILIC after extraction from the Silica gel. As a result, GRN was identified as spot R3 (according to the same retention time compared with GRN standard) and its presence was confirmed by ^{1}H-NMR spectroscopy (B), showing the following corresponding peaks: 5.04 ppm and 3.9-3.4 ppm are characteristic for glucose moiety; 3.05-2.75 ppm and 2.2-1.8 ppm are attributed to the butyl chain in aglycone part of GRN and peak forming a singlet at 2.71 ppm is attributed to the methyl group attached

to the sulfur atom. By analyzing the spot R2 by ^{1}H-NMR, we identified the second major GLS as SBN, according to the characteristic region of peaks for aromatic structure, hydroxylated benzene ring in para position in area of 7.4-6.8 ppm and peaks referred to glucose moiety in area 4,2-3,2 ppm. The purity of GRN was 90% and the purity of SBN 82%. The products contained traces of acetate (1.9 ppm), MeOH (3.3 ppm) and formate (8.45 ppm) (not shown), found by ^{1}H-NMR analysis.

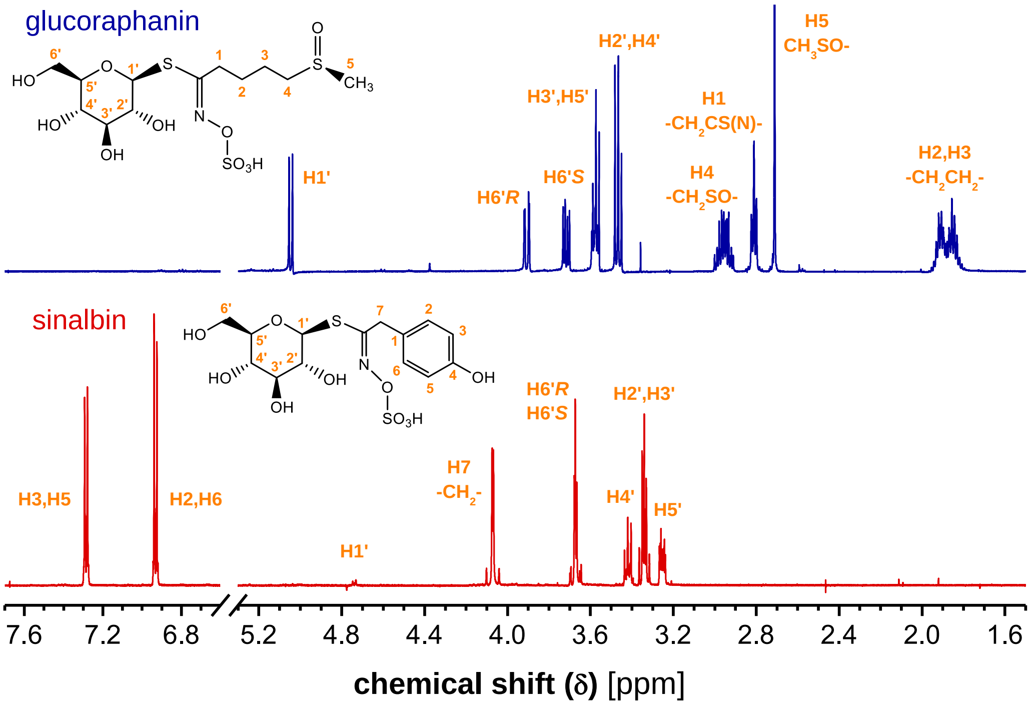

Figure S2. ^{1}H-NMR analysis of GRN and SBN obtained after complete purification.

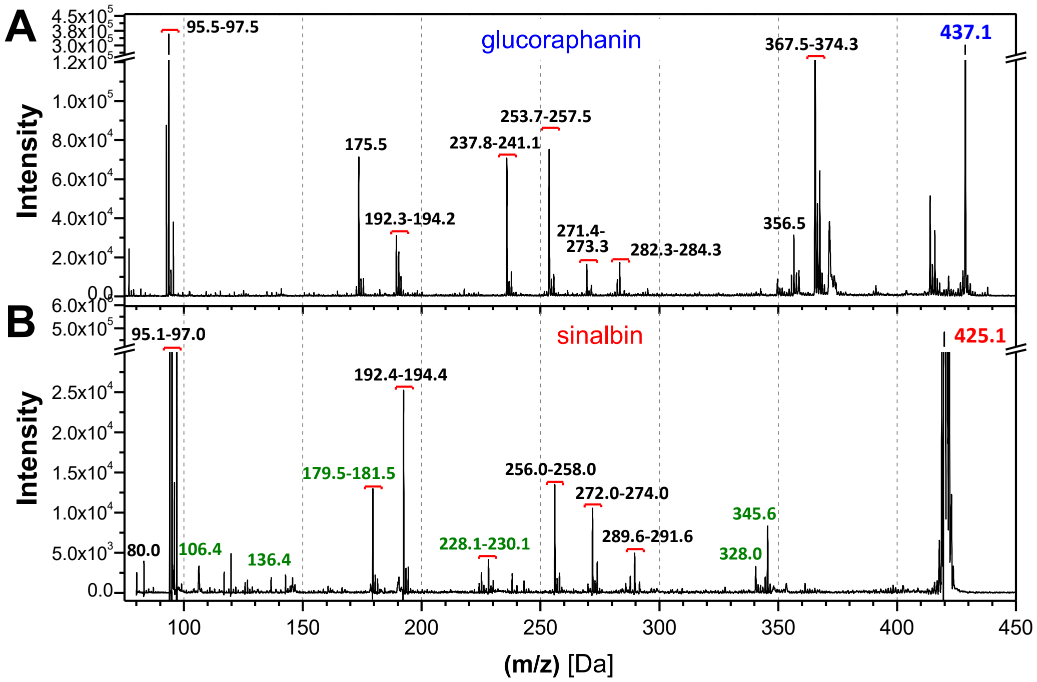

Figure S3. MALDI-TOF/TOF (MS/MS) spectrum of the parental peaks with m/z 437.1 Da (purified GRN) and 425.1 Da (purified SBN). The MS/MS analysis was performed in the negative ion mode with 9-aminoacridine as matrix. Green labelled peaks absent in MS/MS spectrum of GRN (A), represent the fragments originated from the p-hydroxybenzyl aglycone part of the SBN (B).

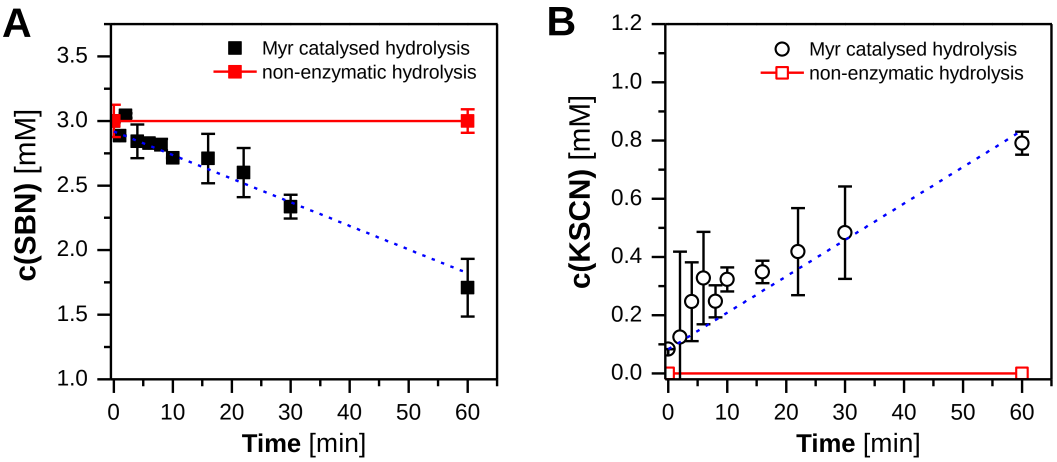

Figure S4. Time course of hydrolysis of SBN (purified from hoary cress) catalyzed by crude Myr preparation from garden cress. The composition of the reaction mixtures was as follows: 300 mM MES-NaOH buffer, pH 6.5, 0.1 mM L-ascorbic acid, 0.3 mg/mL proteins containing active Myr and 3 mM SBN. Aliquots of the reaction mixture were taken at respective time intervals, and reaction in aliquots was stopped by addition of cooled methanol (in ratio 1:2, -20 ˚C). The concentration of SBN estimated by HILIC-based HPLC (see Section 4.5.) and the concentration of KSCN was determined spectrophotometrically (see Section 4.10). The time course of SBN consumption or KSCN formation yields linear dependencies that, by linear regression, result in correlation coefficients of r = −0.935 and r = 0.983. The straight-line slopes were -0.0183 and 0.012 mM/min for SBN consumption and KSCN formation, respectively.

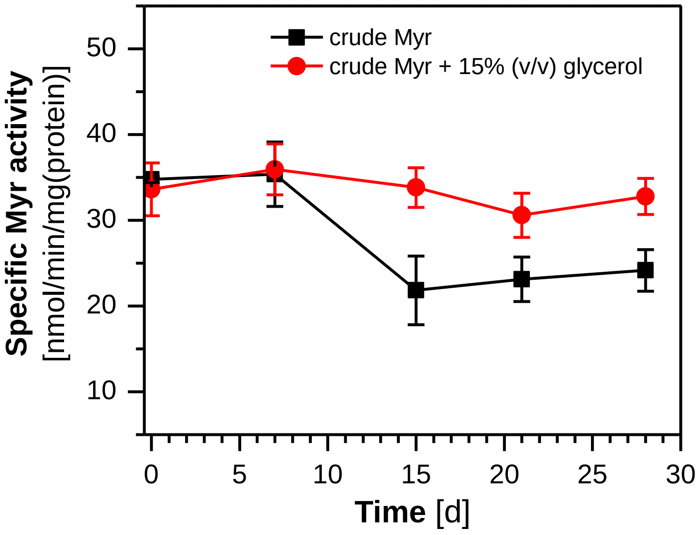

Figure S5. Long-time Myr-stability measurement. The stability of the crude Myr preparate stored in the presence/absence of 15% (v/v) glycerol at 4°C was measured as described in Section 4.8.

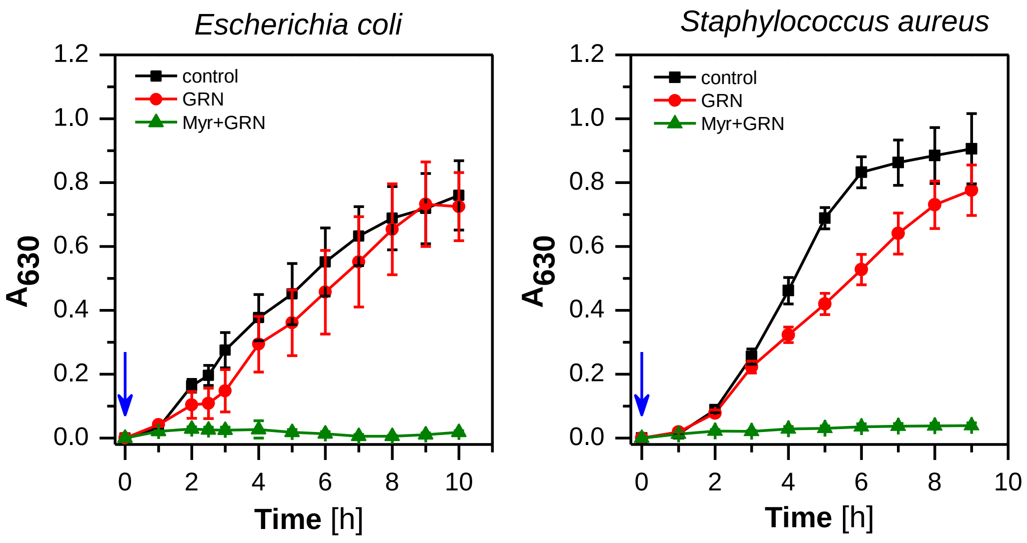

Figure S6. Effect of *in situ* generated SFN on growth of *E. coli* and *S. aureus*. Antibacterial effect of *in situ* formed SFN from GRN by Myr in the presence of L-ascorbate on *E. coli* (CCM 3954) and *S. aureus* (CCM 3953) was monitored until the bacterial growth entered the stationary phase of growth. Reaction mixture (composition of reaction mixture described in section 4.11.) was mixed with the inoculum of the bacterial strains at time 0 h (blue arrow). The crude Myr preparation and L-ascorbate alone had no effect on bacterial growth. GRN slightly inhibited the growth of *S. aureus* but not *E. coli*. The bacterial strains were cultivated at 37°C.
